# Macular perfusion density evaluation in constant and intermittent exotropia by means of optical coherence tomography angiography

**DOI:** 10.1186/s12886-021-02006-1

**Published:** 2021-06-08

**Authors:** Jing Zhai, Wei Fang, Xueting Yu, Xinjie Ye, Lijie Hou

**Affiliations:** Department of Strabismus and Amblyopia, Affiliated Eye Hospital of Wenzhou Medical University, Hangzhou, 310016 Zhejiang Province China

**Keywords:** Retinal microvasculature, Exotropia, Optical coherence tomography angiography

## Abstract

**Background:**

To quantify and compare retinal microvascular features using optical coherence tomography angiography (OCTA) in constant (XT) and intermittent exotropia (IXT).

**Methods:**

An observational cross-sectional study was conducted from September 2020 to November 2020 at the Affiliated Eye Hospital of Wenzhou Medical University. OCTA examination was performed to evaluate the macular perfusion density of the deep capillary plexus (DCP), superficial capillary plexus (SCP), and foveal avascular zone (FAZ) parameters in XT and IXT patients, and in age-matched controls. The study parameters were analyzed.

**Results:**

A total of 76 participants, including 16 (21%) XT patients, 24 (32%) IXT patients, and 36 (47%) controls, were recruited. The exodeviation angle was 39.06 ± 10.38 prism degrees (PD) at near and 43.00 ± 9.74 PD at distance in the XT group, and 27.13 ± 18.28 PD at near and 31.04 ± 18.82 PD at distance in the IXT group. The macular perfusion density of the DCP in 6 × 6-mm scans and the SCP in 3 × 3-mm scans were lower in the deviating eyes than in the fixating eyes of XT patients (*p* < 0.001, *p* = 0.032, respectively). The macular perfusion density of the DCP in the deviating eyes of XT and IXT patients was significantly lower than that of the controls. There was no significant difference in the FAZ parameters among the groups.

**Conclusions:**

In XT patients, OCTA revealed lower macular perfusion density in deviating eyes than in fixating eyes and control eyes. IXT patients showed no difference between the two eyes, but the macular perfusion density of the DCP was lower than that of the controls.

## Background

Intermittent (IXT) and constant exotropia (XT) are two common types of divergent strabismus. The pathophysiology of exotropia has not been fully illustrated. Previously, concomitant strabismic patients with normal visual acuity were generally considered to have normal macular structure. However, recent studies have revealed morphological changes in the macular of strabismic patients, including thickened outer retinal layers in XT patients according to optical coherence tomography (OCT) [1, 2]. Furthermore, known relationships exist between retinal thickness and retinal perfusion in healthy subjects [3, 4]. To date, the involvement of the retinal microvasculature in cases of exotropia has not been well defined. Optical coherence tomography angiography (OCTA), as a noninvasive imaging technique, has been used extensively to quantitatively measure the macular perfusion density of the deep retinal capillary plexus (DCP), the superficial retinal capillary plexus (SCP) and the foveal avascular zone (FAZ) parameters [5, 6]. Therefore, the present study aimed to quantitatively evaluate retinal microvasculature parameters in eyes with XT and IXT as well as in age-matched controls using OCTA.

## Methods

### Subjects

This observational cross-sectional study was conducted at the Affiliated Eye Hospital of Wenzhou Medical University from September 2020 to November 2020. The protocol was approved by the Ethics Committee of the Affiliated Eye Hospital of Wenzhou Medical University. The study was performed according to the tenets of the Declaration of Helsinki for research involving human subjects. Before imaging was conducted, written informed consent was obtained directly from the participants or from the parents or legal guardians of participants younger than 18 years.

The inclusion criteria were patients with IXT and XT presenting to our hospital who needed surgical intervention for strabismus. Strabismic patients enrolled in the study should satisfied the following conditions simultaneously: (1) a best corrected visual acuity (BCVA) of either eye of 20/20 or better, (2) a refractive spherical error < 6.0 diopters (D) and a cylindrical error < 3 D, and (3) no other type of strabismus. Control subjects were recruited from patients who presented at the eye hospital with a BCVA of 20/20 or better, a refractive spherical error < 6 D and a cylindrical error < 3 D, and no evidence of strabismus or ocular abnormality. The exclusion criteria included the presence of nystagmus, amblyopia, paralytic or restrictive exotropia, any organic eye diseases, and a history of strabismus surgery. Patients with neurologic disease or systemic conditions were also excluded.

Participants were divided into three groups: (1) the XT group, (2) the IXT group, and (3) the control group. Furthermore, we performed an additional comparison, deviating eye vs fixating eye, in participants in the XT and IXT groups. In the exotropia groups, the deviating eye was selected as the study eye, and correspondingly, the contralateral eye was defined as the control eye. In the control group, the right eye was collected as the study eye. The deviating eye was identified (1) as the eye that habitually deviated in daily life and (2) via the hole-in-the card method. For the second method, each patient held a test card with a 2-cm diameter circular hole in the center at a distance of an arm length. Patients were asked to open both eyes and look at the distance and near targets through the hole. The deviating eye spontaneously exhibited exodeviation, while the fixating eye looked straight at the target.

All subjects underwent a complete ophthalmological examination, including visual acuity testing, refractive status (spherical equivalent), slit-lamp examination, fundoscopy, ocular exodeviation (using the prism alternate cover test at near (33 cm) and distance (6 m)), eye movement, and OCTA evaluation.

### OCTA scanning data acquisition and processing

**Fig. 1:**
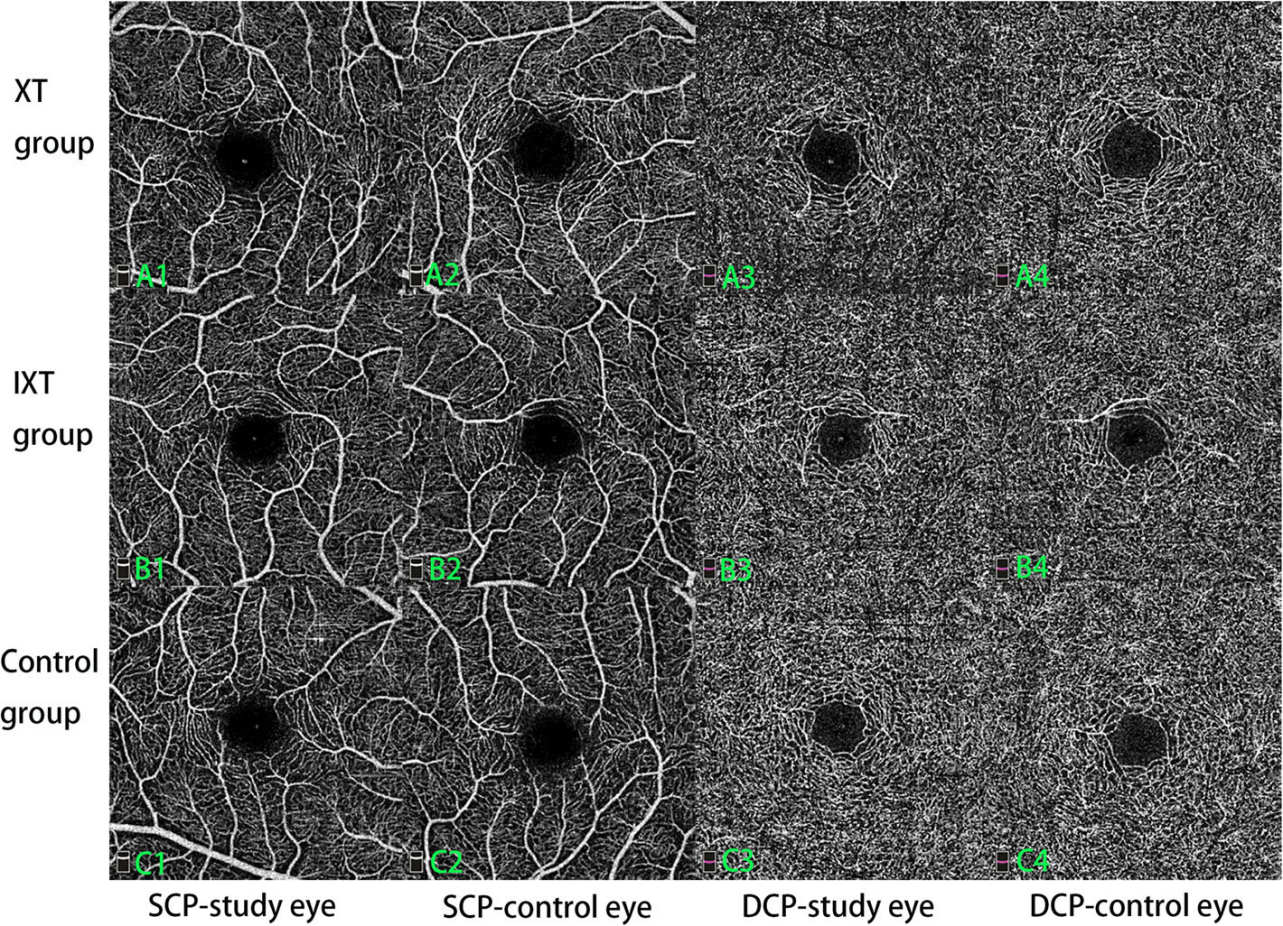
Macular perfusion density of the SCP and DCP from 3 × 3-mm scan. **A**-**C** denote three individuals in XT, IXT and control groups, while number 1–4 denote SCP of the study eye, SCP of the contralateral eye, DCP of the study eye, DCP of the contralateral eye, respectively. (Perfusion density: A1: 45.8%, A2: 51.6%, A3: 48.8, A4: 52.7%, B1: 49.8%, B2: 50.1%, B3: 53.5%, B4: 53.9%, C1: 47.6%, C2: 47.4%, C3: 56.6%, C4: 56.0%). (XT, constant exotropia; IXT, intermittent exotropia; SCP, superficial capillary plexus; DCP, deep capillary plexus)

**Fig. 2:**
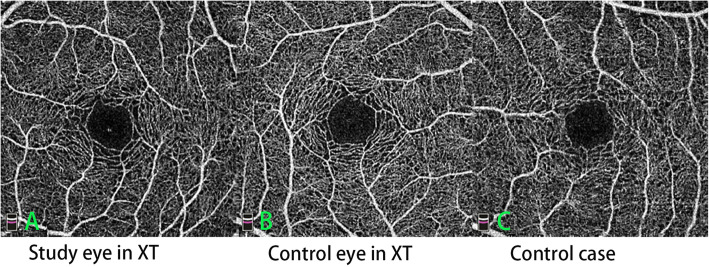
FAZ from 3×3-mm scan in both eyes of a XT patient and a control case. **A** FAZ scan in the study eye of a case in the XT group (area: 0.277 mm^2^, perimeter: 2.012 mm, AI:1.08). **B** FAZ scan in the contralateral eye of the same case (area: 0.256mm^2^, perimeter: 1.931 mm, AI:1.11). **C** FAZ scan in a control individual (area:0.236 mm^2^, perimeter:1.855 mm, AI:1.08). (FAZ, foveal avascular zone; XT, constant exotropia; AI, acircularity index)

OCTA images were obtained using RTVue XR Avanti device (Optovue Inc., Fremont, CA, USA) which used the AngioVue system with the split-spectrum amplitude decorrelation angiography (SSADA) algorithm. Moreover, three dimensional projection artifacts removal algorithms were implemented to solve the issue of projection artifacts in the DCP slab. Both 6 × 6-mm and 3 × 3-mm scans of the macular region were performed for each participant with undilated pupil. The AngioVue software (version:2017.1.0.155) automatically segmented the layers and visualized the SCP and DCP in the OCTA images (the SCP consists of retinal capillaries ranging from the internal limiting membrane to 10 μm above the inner plexiform layer; the DCP extends from 10 μm above the inner plexiform layer to 10 μm below the outer plexiform layer) (Fig. 1). The segmentations of the SCP and DCP in each image were manually inspected and adjusted. FAZ parameters were measured in the 3 × 3-mm scan. FAZ parameters included the FAZ area (Fig. 2), FAZ perimeter, and acircularity index (AI) = measured perimeter/perimeter of the regular circle with the same FAZ area. Macular thickness was measured by crossline module procedures that obtained retinal thickness in the 3 × 3-mm scan from the inner limiting membrane to the retinal pigment epithelium. All images were obtained by a single experienced technician (JW). Images with a signal strength index less than 6 of 10, or unclear layer segmentation were excluded.

### Statistical analysis

Statistical analyses were performed with SAS 9.2 statistical software (SAS Institution Inc., Cary, NC, USA). Categorical variables are shown as numbers and percentages; quantitative variables are presented as the means ± standard deviations or medians (interquartile ranges). The Shapiro-Wilk test (W score) was used to verify the normality of the distribution of quantitative data. Analysis of variance (ANOVA) with the least significant difference (LSD) method was used to analyze the intergroup and intragroup differences. A paired t-test was used to compare the study eye with the contralateral eye. The Spearman correlation test was used to explore the correlation between the exodeviation angle and macular perfusion density. The chi-square or Fisher’s exact test was used to compare categorical data. For all analyses, two-sided *p* value of < 0.05 was considered statistically significant.

## Results

A total of 76 patients were enrolled, including 34 females and 42 males. There were 16 (21%) XT patients, 24 (32%) IXT patients and 36 (47%) control participants recruited. The baseline characteristics of the patients are given in Table 1. Most of the variables were equal distributed among three groups, except the angle of exodeviation (39.06 ± 10.38 prism degrees (PD) at near and 43.00 ± 9.74 PD at distance in the XT group, and 27.13 ± 18.28 PD at near and 31.04 ± 18.82 PD at distance in the IXT group, *p* < 0.001).

**Table 1:** Demographics and refractive status of study subjects

Items	XT group Mean ± SD/ Numbers (%)	IXT group Mean ± SD/ Numbers (%)	Control group Mean ± SD/ Numbers (%)	*p* value
Age (yrs)	12.88 ± 5.82	10.83 ± 5.16	10.19 ± 2.83	0.133
Sex				0.057
Female	4 (25)	9 (37.50)	21 (58.33)	
Male	12 (75)	15 (62.50)	15 (41.67)	
Spherical equivalent, D*	−1.17 ± 1.73	−1.69 ± 2.58	−2.09 ± 1.46	0.283
Angle of deviation, PD				
N	39.06 ± 10.38	27.13 ± 18.28		**< 0.001**
D**	43.00 ± 9.74	31.04 ± 18.82		**< 0.001**

*XT* constant exotropia, *IXT* intermittent exotropia, D* diopter, *PD* prism degree, *N* near, D** distance, *SD* standard deviation; *p* values in bold reached statistical significance

We found a significant decrease in the DCP perfusion density of the study eyes when compared to the contralateral eyes in the XT group in the 6 × 6-mm scans (43.8% ± 3.2% (Mean ± SD) versus 52.4% ± 4.9%, *p* < 0.001). While in the 3 × 3-mm scans, the SCP perfusion density was significant lower in the study eyes than in the contralateral eyes (47.1% ± 1.9% versus 48.8% ± 2.2%, *p* = 0.032). No significant difference between the study eyes and contralateral eyes was found in either the IXT group or the control group (Table 2).

**Table 2:** Comparison of macular vessel density between the study eyes and control eyes in each group

Items	XT group		*p*	IXT group		*p*	Control group		*p*
	Study eye	Control eye		Study eye	Control eye		Study eye	Control eye	
6 × 6-mm OCTA macular scans									
SCP (%, Mean ± SD)	50.38 ± 2.42	51.04 ± 2.44	0.232	50.81 ± 2.45	50.12 ± 2.90	0.216	51.04 ± 2.49	50.73 ± 2.04	0.400
DCP (%, Mean ± SD)	43.81 ± 3.24	52.38 ± 4.86	**< 0.001**	47.22 ± 6.89	49.04 ± 7.27	0.190	51.09 ± 5.30	50.86 ± 4.42	0.805
3 × 3-mm OCTA macular scans									
SCP (%, Mean ± SD)	47.12 ± 1.90	48.77 ± 2.18	**0.032**	47.76 ± 2.82	48.63 ± 2.21	0.055	48.26 ± 2.34	48.20 ± 2.48	0.901
DCP (%, Mean ± SD)	51.22 ± 3.60	52.69 ± 4.10	0.193	51.13 ± 4.19	51.62 ± 3.45	0.471	53.83 ± 2.54	54.15 ± 2.65	0.413
FAZ parameters									
Area (mm^2^)	0.25 ± 0.10	0.26 ± 0.11	0.350	0.27 ± 0.09	0.30 ± 0.09	0.114	0.27 ± 0.10	0.27 ± 0.09	0.653
Perimeter (mm)	1.95 ± 0.43	2.00 ± 0.42	0.389	2.02 ± 0.34	2.09 ± 0.38	0.161	2.05 ± 0.36	1.99 ± 0.45	0.351
AI	1.12 ± 0.03	1.14 ± 0.06	0.225	1.12 ± 0.04	1.13 ± 0.04	0.172	1.12 ± 0.03	1.13 ± 0.06	0.358
Macular thickness (μm, Mean ± SD)	310.00 ± 15.69	307.94 ± 16.18	0.119	312.71 ± 13.60	313.13 ± 14.14	0.638	308.03 ± 12.94	309.22 ± 13.85	0.078

*XT* constant exotropia, *IXT* intermittent exotropia, *OCTA* optical coherence tomography angiography, *SCP* superficial capillary plexus, *DCP* deep capillary plexus, *FAZ* foveal avascular zone, *AI* acircularity index, *SD* standard deviation; *p* values in bold reached statistical significance

In terms of the macular perfusion density of the study eyes among the three groups, we found a significant decrease of DCP perfusion density in the XT and IXT groups when compared to their counterparts in the control group both in 3 × 3-mm and 6 × 6-mm scans, however, the difference between the XT group and IXT group was not significant. Besides, there was also no significant difference in the macular perfusion density of the SCP of the study eyes among the three groups (Table 3).

**Table 3:** Macular microstructure of the study eyes in the three groups

Items	XT vs Control group	IXT vs Control group	XT vs IXT group
	(95% CI)	(95% CI)	(95% CI)
6 × 6-mm OCTA macular scans			
SCP (%,Mean ± SD)	−0.66 (− 2.14, 0.81)	− 0.23 (− 1.56, 1.10)	−0.43 (− 2.05, 1.18)
DCP (%,Mean ± SD)	−7.28* (− 10.57, −3.99)	−3.86* (−6.83, − 0.90)	−3.42 (− 7.01, 0.18)
3 × 3-mm OCTA macular scans			
SCP (%,Mean ± SD)	− 1.14 (− 2.59, 0.31)	− 0.49 (− 1.77, 0.78)	−0.64 (− 2.20, 0.91)
DCP (%,Mean ± SD)	−2.61* (− 4.62, − 0.60)	−2.70* (− 4.46, − 0.93)	0.09 (− 2.07, 2.24)
FAZ parameters			
Area (mm^2^)	−0.032 (− 0.084, 0.025)	−0.006 (− 0.055, 0.042)	−0.024 (− 0.084, 0.035)
Perimeter (mm)	− 0.099 (− 0.320, 0.121)	−0.028 (− 0.221, 0.166)	−0.071 (− 0.308, 0.165)
AI	0.005 (− 0.015, 0.024)	0.002 (− 0.015, 0.019)	0.003 (− 0.018, 0.024)
Macular thickness (μm, Mean ± SD)	1.972 (− 6.266, 10.210)	4.681 (− 2.545, 11.906)	−2.708 (− 11.557, 2.545)

*XT* constant exotropia, *IXT* intermittent exotropia, *OCTA* optical coherence tomography angiography, *SCP* superficial capillary plexus, *DCP* deep capillary plexus, *FAZ* foveal avascular zone, *AI* acircularity index, *SD* standard deviation, *CI* confidential interval; *indicates *p* < 0.05

Neither intra-group nor inter-group differences were found for the macular thickness or FAZ parameters in the 3 × 3-mm scan, including the FAZ area, perimeter, and acircularity index (Tables 2 and 3).

## Discussion

IXT and XT patients commonly present with a deviating preference. The deviating eye transmits “misaligned” optical stimulation, which is projected to the noncorresponding point, while visual images are projected to the fovea of the fixating eye. This phenomenon can cause suppression or potentially abnormal retinal correspondence [7–11]. In reports of strabismic patients with abnormal binocular vision development, OCT exhibited changes in macular retinal thickness [1]. Retinal thickness was correlated with retinal perfusion for both healthy subjects and amblyopia [3, 4]. Amblyopic eyes showed a thicker foveola than that of visually normal control eyes [12], and lower macular vessel density than that of the companion eyes and the controls [13–16]. Recently, Chen et al.revealed the relationship between retinal thickness and retinal vessel changes in the amblyopia eyes, showing SCP vessel density was positively correlated with inner retinal thickness in the fovea and parafovea [17]. In strabismus patients, recent studies have revealed thickened outer retinal layers using OCT in XT patients [2]. Here, we examined whether abnormal retinal microvasculature existed in addition to retinal thickness changes in exotropia patients. In our study, when both eyes (deviating vs fixating) of XT patients were analyzed as a self-control, OCTA illustrated a statistically significant decrease in the macular perfusion density in the deviating eye. This finding may be related to the constant lack of normal visual information received by the deviating eye, although we could not differentiate whether the finding was the cause or the consequence of the constant exodeviating status. However, when both eyes of IXT patients were compared, there was no statistically significant difference in macular perfusion density or macular thickness. In IXT patients, who can control the deviating eye well and for whom spontaneous exodeviation may only manifest in some states, such as fatigued or ill, the change in retinal morphology was rarely due to the same optical stimulation received in both eyes. Further study is needed to determine whether correlation exists between these subtle retinal microvascular changes and the corresponding segmented retinal thickness or binocular vision function.

A statistically significant decrease was observed in the macular perfusion density of the DCP in the study eyes of XT and IXT patients when compared with those of normal human controls. The DCP is a plexus below the inner nuclear layer that is supplied by vertically oriented interconnecting vessels from the superficial vascul [18, 19]. Due to the presence of the terminal anastomotic capillary networks, in addition to its local metabolic needs, the DCP itself may be vulnerable to ischemia or hypoxic injury [20, 21].

According to the study analysis results, the exodeviation angle was not correlated with macular perfusion density in the strabismic group either near or distance. These findings are in concordance with those of a previous study showing no association between topographical changes in the macular and the angle of deviation in concomitant strabismic patients [2]. Jampolsky postulated that XT may evolve from IXT due to gradual suppression and decompensate [7]. However, in our study, we could not find a decreasing trend in macular perfusion density from IXT to XT. Involvement of the retinal microvasculature in the progression from IXT to XT could not be identified. Also, more recently, Inal et al. speculated that after surgery there was a significant increase in measurements of the SCP and DCP vessel density for operated eyes. There were no significant changes for the fellow non-operated eye [22]. It would be interesting to observe whether there are changes in macular perfusion after surgery, and to explore some potential factors affecting the macular perfusion changes.

This study has several limitations. First, the sample size was relatively small in the study. Second, as most patients here had a relatively large angle of exodeviation and required strabismus surgery, whether the findings hold true for exophoria or a small angle of deviation is thus far unclear. Further studies using a larger sample size are needed, including the evaluation of the macular intra-retinal layer thicknesses and the perfusion change after surgery. Additional studies will be integrated with our findings and reveal their clinical relevance.

## Conclusions

On OCTA, patients with XT demonstrated lower macular perfusion densities in the SCP and DCP of the deviating eyes than of the fixating eyes, as well as lower perfusion densities than control individuals. IXT patients showed no retinal perfusion density difference between the two eyes, but their DCPs in the deviating eyes had a lower perfusion density than those of control individuals. Further studies are needed to confirm the current results in our study and to reveal the clinical relevance of this finding.

## Data Availability

The datasets used and/or analysed during the current study are available from the corresponding author on reasonable request.

## Abbreviations

OCTA: Optical coherence tomography angiography
IXT: Intermittent exotropia
XT: Constant exotropia
SCP: Superficial capillary plexus
DCP: Deep capillary plexus
FAZ: Foveal avascular zone
AI: Acircularity index
OCT: Optical coherence tomography
BCVA: Best corrected visual acuity
CI: Confidence interval
D*: Diopter
PD: Prism degree
N: Near
D**: Distance
SD: Standard deviation
SSADA: Split-spectrum amplitude decorrelation angiography
ANOVA: Analysis of variance
LSD: Least significant difference

## References

[1] Oka M, Yamashita T, Ono S, Kubo I, Tabuchi A (2013). Quadrantal macular retinal thickness changes in strabismus subjects with abnormal binocular vision development. Jpn J Ophthalmol.

[2] Wen Y, Yan J, Wang Z, Shen T, Qiu X, Deng D (2020). Topographical profiles of macula and optic nerve head in concomitant strabismus patients as measured using OCT and CSLO. Graefes Arch Clin Exp Ophthalmol.

[3] Yu J, Gu R, Zong Y, Xu H, Wang X, Sun X (2016). Relationship between retinal perfusion and retinal thickness in healthy subjects: an optical coherence tomography angiography study. Invest Ophthalmol Vis Sci.

[4] Cheung CY, Li J, Yuan N, Lau GYL, Chan AYF, Lam A (2019). Quantitative retinal microvasculature in children using swept-source optical coherence tomography: the Hong Kong children eye study. Br J Ophthalmol.

[5] Wang Q, Chan S, Yang JY, You B, Wang YX, Jonas JB (2016). Vascular density in retina and choriocapillaris as measured by optical coherence tomography angiography. Am J Ophthalmol.

[6] Iafe NA, Phasukkijwatana N, Chen X, Sarraf D (2016). Retinal capillary density and foveal avascular zone area are age-dependent: quantitative analysis using optical coherence tomography angiography. Invest Ophthalmol Vis Sci.

[7] Jampolsky A (1954). Differential diagnostic characteristics of intermittent exotropia and true exophoria. Am Orthopt J.

[8] Serrano-Pedraza I, Manjunath V, Osunkunle O, Clarke MP, Read JC (2011). Visual suppression in intermittent exotropia during binocular alignment. Invest Ophthalmol Vis Sci.

[9] Cooper J, Record CD (1986). Suppression and retinal correspondence in intermittent exotropia. Br J Ophthalmol.

[10] Cooper J, Feldman J, Pasner K (2000). Intermittent exotropia: stimulus characteristics affect tests for retinal correspondence and suppression. Binocul Vis Strabismus Q.

[11] Evans BJW (2002). Pickwell's binocular vision anomalies: investigation and treatment.

[12] Li J, Ji P, Yu M (2015). Meta-analysis of retinal changes in unilateral amblyopia using optical coherence tomography. Eur J Ophthalmol.

[13] Lonngi M, Velez FG, Tsui I, Davila JP, Rahimi M, Chan C (2017). Spectral-domain optical coherence tomographic angiography in children with amblyopia. JAMA Ophthalmol.

[14] Yilmaz I, Ocak OB, Yilmaz BS, Inal A, Gokyigit B, Taskapili M (2017). Comparison of quantitative measurement of foveal avascular zone and macular vessel density in eyes of children with amblyopia and healthy controls: an optical coherence tomography angiography study. J AAPOS.

[15] Araki S, Miki A, Goto K, Yamashita T, Yoneda T, Haruishi K (2019). Foveal avascular zone and macular vessel density after correction for magnification error in unilateral amblyopia using optical coherence tomography angiography. BMC Ophthalmol.

[16] Pujari A, Chawla R, Mukhija R, Obedulla H, Phuljhele S, Saxena R (2019). Assessment of macular vascular plexus density using optical coherence tomography angiography in cases of strabismic amblyopia. Indian J Ophthalmol.

[17] Chen W, Lou J, Thorn F, Wang Y, Mao J, Wang Q (2019). Retinal microvasculature in amblyopic children and the quantitative relationship between retinal perfusion and thickness. Invest Ophthalmol Vis Sci.

[18] Stone J, van Driel D, Valter K, Rees S, Provis J (2008). The locations of mitochondria in mammalian photoreceptors: relation to retinal vasculature. Brain Res.

[19] Tan PE, Yu PK, Balaratnasingam C, Cringle SJ, Morgan WH, McAllister IL (2012). Quantitative confocal imaging of the retinal microvasculature in the human retina. Invest Ophthalmol Vis Sci.

[20] Campbell JP, Zhang M, Hwang TS, Bailey ST, Wilson DJ, Jia Y (2017). Detailed vascular anatomy of the human retina by projection-resolved optical coherence tomography angiography. Sci Rep.

[21] Nemiroff J, Kuehlewein L, Rahimy E, Tsui I, Doshi R, Gaudric A (2016). Assessing deep retinal capillary ischemia in paracentral acute middle maculopathy by optical coherence tomography angiography. Am J Ophthalmol.

[22] Inal A, Yilmaz I, Ocak OB, Aygit ED, Celik S, Pasaoglu I (2019). Optical coherence tomography angiography: are there any changes in measurements afer strabismus surgery?. J Pediatr Ophthalmol Strabismus.

